# Clinical Utility of Exome Sequencing and Reinterpreting Genetic Test Results in Children and Adults With Epilepsy

**DOI:** 10.3389/fgene.2020.591434

**Published:** 2020-12-18

**Authors:** Yong-li Jiang, Changgeng Song, Yuanyuan Wang, Jingjing Zhao, Fang Yang, Qiong Gao, Xiuxiu Leng, Yulin Man, Wen Jiang

**Affiliations:** Department of Neurology, Comprehensive Epilepsy Center, Xijing Hospital, Fourth Military Medical University, Xi’an, China

**Keywords:** genetic testing, next-generation sequencing, reinterpretation, epilepsy, adults

## Abstract

The clinical utility of genetic testing for epilepsy has been enhanced with the advancement of next-generation sequencing (NGS) technology along with the rapid updating of publicly available databases. The aim of this study was to evaluate the diagnostic yield of NGS and assess the value of reinterpreting genetic test results in children and adults with epilepsy. We performed genetic testing on 200 patients, including 82 children and 118 adults. The results were classified into three categories: positive, inconclusive, or negative. The reinterpretation of inconclusive results was conducted in April 2020. Overall, we identified disease-causing variants in 12% of the patients in the original analysis, and 14.5% at reinterpretation. The diagnostic yield for adults with epilepsy was similar to that for children (11 vs. 19.5%, *p* = 0.145). After reinterpretation, 9 of the 86 patients who initially had inconclusive results obtained a clinically significant change in diagnosis. Among these nine revised cases, five obtained positive diagnoses, representing a diagnosis rate of 5.8% (5/86). Manual searches for additional evidence of pathogenicity for candidate variants and updated patient clinical information were the main reasons for diagnostic reclassification. This study emphasizes the diagnostic potential of combining NGS and reinterpretation of inconclusive genetic test reports in children and adults with epilepsy.

## Introduction

Epilepsy is a common and highly heterogeneous neurological disease, with an estimated prevalence of 6 per 1,000 in the general population (Fiest et al., 2017). Genetic factors have long been recognized as playing an important role in the development of this disorder, a rationale supported by family studies and the identification of specific epilepsy syndrome-causing variants (Myers and Mefford, 2015; Perucca et al., 2020). Moreover, it is now thought that more than 70% of epilepsy cases have a genetic basis in both children and adults (Myers et al., 2019; Scala et al., 2020). Consequently, genomic testing of patients with epilepsy is becoming increasingly routine in clinical practice, given that detecting genetic causes may provide accurate prognoses and optimize the management and treatment options for some epileptic patients. However, the phenotypes associated with epilepsy syndromes are often variable and unspecific, involving several genes, while specific genetic variants are frequently associated with a wide phenotypic spectrum (Zhu et al., 2014). This considerable heterogeneity makes it challenging to precisely identify the underlying genetic cause of most epilepsies.

The emergence of next-generation sequencing (NGS) technologies, such as targeted gene panels, whole-exome sequencing (WES), and whole-genome sequencing (WGS), has revolutionized the application of genetic testing and made it technically practicable to evaluate hundreds of genes in a single test (Symonds and McTague, 2020). Over the past decades, NGS approaches have led to the identification of several epilepsy-related genes and expanded the knowledge of phenotypes associated with known genes (Dunn et al., 2018). The reported diagnostic yield of NGS for patients with epilepsy ranges from 10 to 40%, depending on the method of analysis used and the phenotypes among the studied cohorts (Butler et al., 2017; Mei et al., 2017; Perucca et al., 2017). However, although NGS has the potential to improve the detection of disease-causing variants, data interpretation remains the main challenge, especially for variants of uncertain significance, given our incomplete knowledge about the function of individual disease-causing variants and genes (Duzkale et al., 2013; Kassahn et al., 2014; Seaby and Ennis, 2020).

The reinterpretation of genetic data has proven to be an effective means of revealing new disease-causing variants and can increase the diagnostic yield (Wenger et al., 2017). Indeed, the American College of Medical Genetics and Genomics (ACMG) has proposed that previously reported genetic variants be periodically reviewed (Richards et al., 2015; Deignan et al., 2019). Thus, owing to the rapid advances in publicly available databases and the ongoing updating of the clinical phenotypes of the patients, it is particularly important in epilepsy to periodically reinterpret genetic test reports (Epilepsy Genetics Initiative, 2019; Rochtus et al., 2020). Here, we aimed to evaluate the clinical utility and diagnostic potential of reinterpreting NGS results in a cohort of 200 pediatric and adult epileptic patients.

## Materials and Methods

### Patients

From December 2015 to February 2019, a total of 200 patients were recruited sequentially from the Department of neurology of the Comprehensive Epilepsy Center in Xijing Hospital, a tertiary academic hospital in Shaanxi Province, China. For an etiological diagnosis, 80 patients were tested using a commercially available gene panel and 120 patients were tested by WES. The demographic and clinical characteristics, seizure history, EEG findings, brain imaging reports, and antiepileptic drug (AED) medications used were collected and summarized. Epilepsy syndromes and types were classified according to the consensus proposed by the International League Against Epilepsy (ILAE) (Scheffer et al., 2017). Familial history was defined as a history of epilepsy in first-degree relatives. Informed consent was obtained from the patients and their parents/legal guardians. This study was approved by the ethics committee of Xijing Hospital and conducted in agreement with the relevant guidelines and regulations.

### Exome Sequencing and Bioinformatics Analysis

Genomic DNA was extracted from peripheral blood. The exome was captured using either the GenCap custom enrichment kit (including 153 epilepsy-related genes; MyGenostics Inc., Beijing, China, Supplementary Table 1) or SureSelect XTHuman All Exon v4 (Agilent Technologies, Santa Clara, CA, United States) following the manufacturers’ protocols. The enriched libraries were sequenced on the Illumina HiSeq X Ten sequencer (Illumina, San Diego, CA, United States) with a paired-end read length of 150 bp. For the two methods, the average on targeted sequencing depth was 334.50× with 95.71% regions were covered at greater than 20×, and was 160.11× with 97.41% regions were covered at greater than 20×, respectively (Supplementary Table 2). Raw image files were processed using Bcl2Fastq conversion software (Bcl2Fastq 2.18.0.12, Illumina) or cutadapt v1.16 for base calling and raw data generation. Clean reads were aligned to the reference human genome (hg19) using BWA v0.7.15 or v0.7.12-r1044. Duplicated reads were removed using the Picard program, and SNP and indel variants were detected using GATK v3.7 or v4.0. The identified variants were annotated using Exome-assistant or ANNOVAR^1^. MagicViewer or IGV (Integrative Genomics Viewer) v2.8.2 was used to view short-read alignments and confirm the candidate SNPs and indels.

In this study, we analyzed exome sequence data from 200 families: 193 non-trios (proband only; 80 were tested using a panel and 113 were tested by WES); 7 trios (proband and both parents). For the seven trios, paternity/maternity was checked by a freely available software package, KING^2^ using NGS data. For the 193 non-trios, paternity/maternity were detected by short tandem repeats (STR) analysis.

### Variant Prioritization and Interpretation

Variants were filtered and prioritized based on the following parameters: (i) Variant reads should be more than 5 and the mutation ratio should be no less than 30%; (ii) the minor allele frequency (MAF) should be <1% in several databases, such as dbSNP138, 1000 Genomes, and ESP6500; and should not be present in the InNormal database (MyGenostics); (iii) conservation using GERP++, and pathogenicity prediction from Mutation Taster, SIFT, and PolyPhen; (iv) if the variants were synonymous and they were reported in HGMD or ClinVar, left them; and (v) association(s) with human neurological disease (s) (for WES data). Variants were further filtered based on genotype and possible inheritance models. The filtered variants were reviewed according to the recommendation of the ACMG (Richards et al., 2015) and were considered pathogenic based on the phenotype and family history of the patients. All putative causative variants identified by NGS were confirmed by conventional Sanger sequencing. To detect the inheritance status, segregation analyses of variants with the phenotype were performed in the family members of the probands who were available for molecular screening.

### Genetic Reports Classifications

The genetic results were classified into three categories: (i) positive results, where a clinically significant variant (pathogenic or likely pathogenic) identified in a gene could completely explain the phenotypes of a case; (ii) negative results, where no variants were initially reported in the genetic test or where variants were classified as likely benign/benign after reinterpretation; (iii) inconclusive results [all other results not in categories i and ii, including genetic results reported variants of unknown significance (VUS)].

### Reinterpretation of Genetic Reports

Repeat interpretation was conducted by removing cases with either a positive genetic diagnosis or a negative result from the overall cohort in April 2020. Reinterpretation was performed based on updated information in databases for population frequency (Exome Aggregation Consortium, 1000 Genomes) or clinical association (Human Gene Mutation Database, ClinVar), manually reviewing the literature, and additional phenotypes of the patients for which the reports were inconclusive.

### Statistics Analysis

Categorical variables were expressed as counts and percentages. Continuous variables were summarized using medians and interquartile ranges (IQRs). Chi-square tests with Bonferroni correction or Fisher’s exact test were used to evaluate the proportions of diagnostic results among categorical variables. Two-tailed *p* < 0.01 were considered statistically significant. Statistical analysis was performed using IBM SPSS for Windows v.26.0 (IBM, New York, United States).

## Results

### Characteristics of the Patients

Our cohort consisted of 200 patients with genetically unexplained epilepsy. Among them, 82 (41%) were children and 118 (59%) were adults, with 104 males (52%) and 96 females (48%) (sex ratio M:F = 1.1:1). The mean age was 15.5 years (*SD* = ±4.7, median = 15 years) and ranged from 2 to 50 years at testing. Generalized epilepsy was reported in 101 cases (50.5%), focal epilepsy in 49 patients (24.5%), combined generalized and focal epilepsy in 41 patients (20.5%), while unknown epilepsy type was reported for 9 patients (4.5%). The median age at seizure onset was 12 years (IQR, 6.3–15 years). Thirty-four (17%) of the participants had some degree of delay or cognitive impairment, including developmental delay, learning disability, speech delay, and/or behavioral issues (referred to as seizure plus). In total, 21.5% of the patients (43/200) had a family history of epilepsy.

### Molecular Results and Stratification of Diagnostic Yield

**FIGURE 1:**
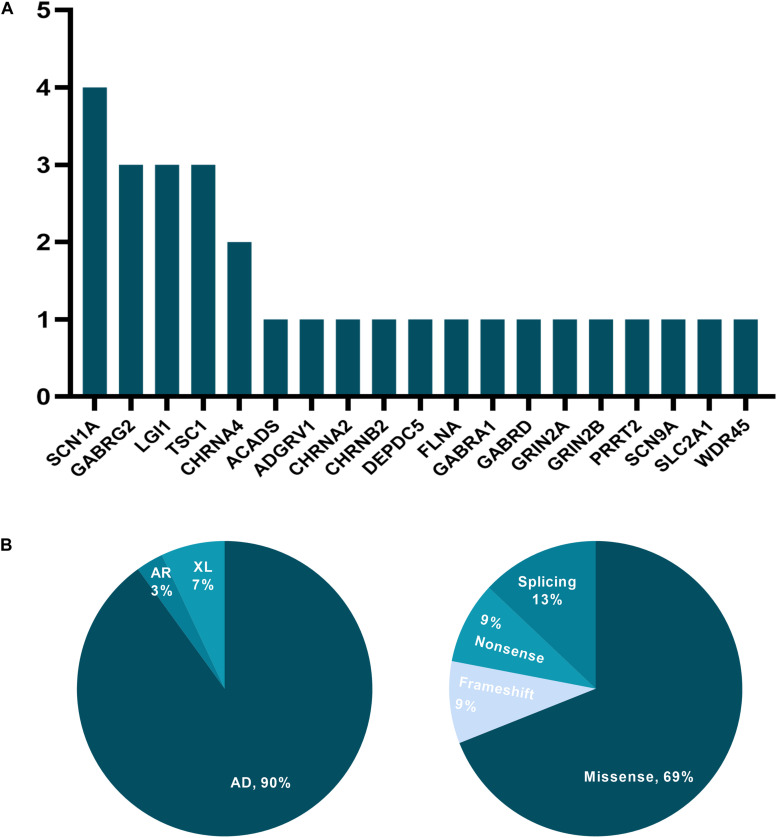
The distribution of mutated genes, mode of inheritance, and variant type for cases with a positive molecular diagnosis. **(A)** *y*-axis: the number of patients; *x*-axis: genes carrying presumed pathogenic variants. **(B)** AD, autosomal dominant; AR, autosomal recessive; XL, X-linked disorders.

Over the 52 months and after reinterpretation, 29 of the initial 200 patients (14.5%) obtained a positive molecular diagnosis, with 31 variants spanning 19 genes being identified (Supplementary Table 3). Testing in 77 patients (38.5%) yielded inconclusive findings, and 94 patients (47%) had negative results. Genes identified in more than one patient who had obtained a positive result were *SCN1A* (*n* = 4), *GABRG2* (*n* = 3), *LGI1* (*n* = 3), *TSC2* (*n* = 3), and *CHRNA4* (*n* = 2), accounting for 26.3% (5/19) of all mutated genes in our study (Figure 1A). The distribution of mode of inheritance and variant type is presented in Figure 1B. Among the 26 autosomal dominant cases, 16 (61.5%) were inherited, and 10 (38.5%) were linked to a *de novo* variant. Two cases were associated with X-linked dominant disorders, one carrying a *de novo* variant and the other an undetermined variant. And one patient diagnosed with autosomal recessive disorders was compound heterozygotes.

The diagnostic yield varied significantly according to age at seizure onset and neurodevelopmental phenotype. The positive rate was highest in patients under than 6 years of age at seizure onset (14/50, 28%), followed by those aged 6–18 years (14/122, 11.5%), contributing 14% to the overall diagnostic yield in the cohort. Furthermore, in our study, patients with seizure plus were significantly more likely to have a positive result (35.3%, *p* = 0.000). However, there was no significant difference in yield between children and adults with epilepsy (19.5 vs. 11%, *p* = 0.145). Additionally, the test sensitivity for the detection of the relevant genetic information was not significantly different (*p* = 0.673) between probands tested with the epilepsy gene panel (13/80, 16.3%) and those tested using WES (16/120, 13.4%) (Table 1).

**TABLE 1:** Testing outcomes stratified by the clinical characteristics of the cohort.

Cohort	Number of patients (% category)				
	With positive result (29)	With inconclusive result (77)	With negative result (94)	Total (% of the whole cohort)	*p*-value
Age at testing (years)					
<18	16 (19.5)	33 (40.2)	33 (40.3)	82 (41.0)	0.145
≥18	13 (11.0)	44 (37.3)	61 (51.7)	118 (59.0)	
Age at seizure onset (years)					
≤6	14 (28.0)	19 (38.0)	17 (34.0)	50 (25.0)	0.000
6–18	14 (11.5)	53 (43.4)	55 (45.1)	122 (61.0)	
≥18	1 (3.6)	5 (17.8)	22 (78.6)	28 (14.0)	
Genetic test module					
Gene panel	13 (16.3)	28 (35.0)	39 (48.7)	80 (40.0)	0.673
WES	16 (13.4)	49 (40.8)	55 (45.8)	120 (60.0)	
Phenotype					
Seizure	17 (10.2)	64 (38.6)	85 (51.2)	166 (83.0)	0.000
Seizure plus^†^	12 (35.3)	13 (38.2)	9 (26.5)	34 (17.0)	
Type of epilepsy					
Focal	12 (24.5)	18 (36.7)	19 (38.8)	49 (24.5)	0.067^a^
Generalized	8 (7.9)	43 (42.6)	50 (49.5)	101 (50.5)	
Combined	9 (21.9)	12 (29.3)	20 (48.8)	41 (20.5)	
Unknown	0	4 (44.4)	5 (55.6)	9 (4.5)	

**^†^Participants had some degree of delay or cognitive impairment, including developmental delay, learning disability, speech delay, and/or behavioral issues. ^a^Fisher’s exact test.**

### Molecular Results After the Initial Analysis

After the first analysis, 24/200 patients (12%) received a positive result, with 25 variants in 17 genes being detected and classified as pathogenic or likely pathogenic (Supplementary Table 3). Ninety patients (45%) had negative results and 86 (43%) obtained inconclusive yields. Additionally, 3 patients with no etiological diagnosis harbored a medically actionable secondary finding in one of the 59 ACMG genes (Supplementary Table 4).

### Repeat Interpretation Findings

**FIGURE 2:**
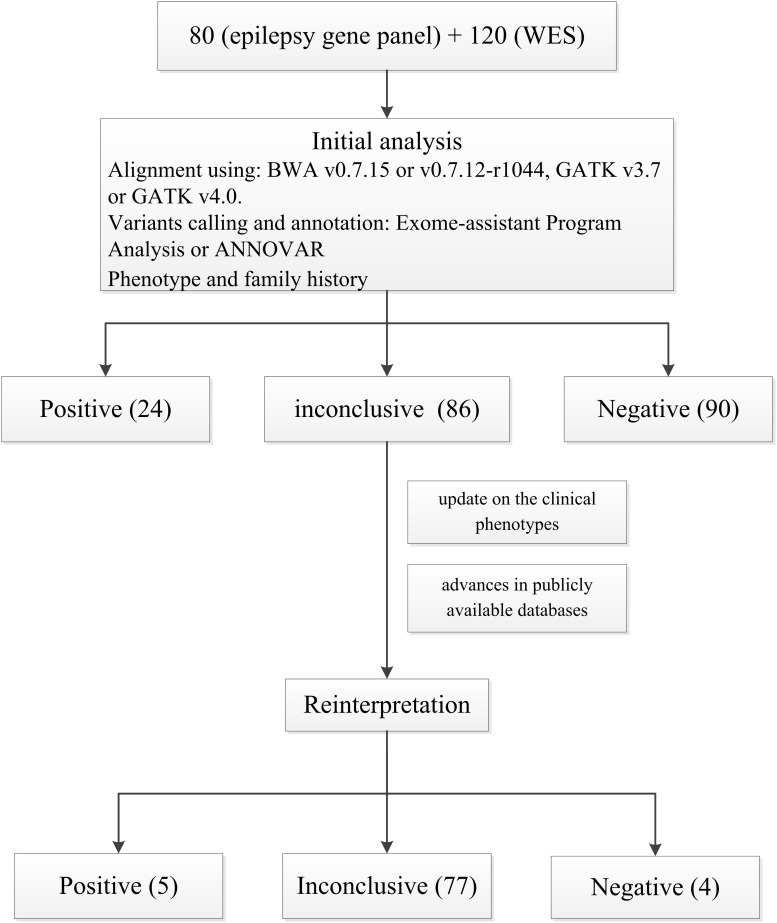
Flow chart of the initial analysis and reinterpretation.

The reinterpretation was done between 14 and 52 months after the report issue date (median 42 months). The reinterpretation of these 86 inconclusive results led to 9 patients (9/86, 10.5%) having a clinically significant change in diagnosis (Table 2 and Figure 2). The second interpretation allowed the identification of five disease-causing variants in 5/86 patients, thereby leading to an additional diagnostic yield of 5.8% (Supplementary Table 3). Three diagnoses resulted from an internal analysis of new disease–gene associations, in which *GABRG2* linked to sleep-related hypermotor epilepsy (SHE, we have performed *in vitro* experiments to investigate these *GABRG2* variants’ function but the results have not yet been published). The result of the genetic testing report for patient P5 diagnosed with Rolandic epilepsy (RE), was upgraded to positive based on a recent publication: the author identified the same *GRIN2A* variant (c.1341T > A p.Asn447Lys) in a boy with RE by WES and demonstrated that Asn447Lys is a gain-of-function variant (Xu et al., 2018). Moreover, an incomplete penetrance of *GRIN2A* variants associated with RE has been extensively reported (Lemke et al., 2013). Therefore, we reclassified this variant as likely pathogenic based on manual adjustment of ACMG guidelines. For patient W85, who was diagnosed as having genetic epilepsy with febrile seizures plus (GEFS+), a missense variant was detected in *SCN9A* (c.5231A > G, p.Tyr1744Cys). This variant was inherited from the patient’s mother who suffered from febrile seizures between the ages of 4–7. Through follow-up, we identified the same variant in the patient’s younger sister who was also diagnosed with GEFS+. Moreover, several recent studies supported a causal role of this gene in epilepsy based on case-level data, particularly in GEFS+ (Mulley et al., 2013; Zhang et al., 2020). Therefore, we considered that this variant may be regarded as pathogenic variant in this pedigree and reclassified the result as positive (see Table 2 for additional details). However, given that *SCN9A* has limited disease-association per the clingen epilepsy working group^3^, the specific functional experiments considering this variant are needed to provide additional supports.

**TABLE 2:** Clinical and genetic characteristics of the patients who obtained a clinically significant change in diagnosis after reinterpretation.

Case	Phenotype	Gene	Variants	References	Inheritance and zygosity	Initial classification	Reinterpretation	Date of initial report	Date of evidence of interest	Reason for reclassification	ACMG
P5	RE (CPS + sGTCS; onset 8 years; intractable on LEV + VPA), centro-temporal spikes on EEG	GRIN2A	NM_000833 c.1341T > A p.Asn447Lys	Xu et al., 2018	AD, maternal, het	Inconclusive	Positive	03/2016	09/2017	Manual search	P (PS3, PS4, PM1, PM2, PP4, BP4)
P20	GTCS + MS (onset 17 years; seizure-free for 5 years on VPA), sharp wave at the frontal and temporal lobe on EEG	RELN	NM_005045.3 c.3712A > C p.Asn1238His	N	AD, paternal, het	Inconclusive	Negative	12/2015	12/2019	ClinVar	B (BS1, BS2, BP4, BP6)
P25	RE (GTCS + CPSCPS; onset 13 years; seizure-free for 4 years on OXC), centro-temporal spikes on EEG				AD, paternal, het			02/2016			
P45	GTCS (onset 2 years; seizure-free for 3 years on TPM)	DEPDC5	NM_001242897 c.2858C > A p.Pro953His	N	AD, paternal, het	Inconclusive	Negative	07/2016	12/2019	ClinVar; 1000Gen	B (BS1, BS2, BP4)
P72	SHE (onset 7 years; intractable on OXC), ID	GABRG2	NM_198903 c.1070C > A p.Thr357Asn	N	AD, *de novo*, het	Inconclusive	Positive	10/2016	01/2019	Manual search	LP (PS2, PM1, PM2, PP3)
W48	SHE (onset 12 years; seizure-free for 2 years on CBZ), rare sharp-slow wave at the forehead during sleep	GABRG2	NM_198903 c.649C > T p.Gln217X	N	AD, paternal, het	Inconclusive	Positive	07/2017		Manual search	LP (PVS1, PM2)
W51	SHE (onset 7 years; some improvement on CBZ), 3.5–4 Hz spike-slow wave at the forehead and anterior temporal	GABRG2	NM_198903 c.269C > T p.Thr90Met	N	AD, maternal, het	Inconclusive	Positive	01/2018		Manual search	LP (PM1, PM2, PP3, PP5)
W53	GTCS (onset 13 years; seizure-free for 5 years on LEV + DPK), spike-slow complex wave at the left frontal lobe on EEG	SCN3A	NM_006922 c.1687C > T p.Arg563Cys	N	AD, paternal, het	Inconclusive	Negative	03/2018	01/2020	ClinVar; follow-up and updated family history	LB (PP3, BS4, BP1)
W85	GTCS (onset 8 years, intractable on LEV), this variant was also identified in the patient’s sister who had GEFS+	SCN9A	NM_002977.3 c.5231A > G p.Tyr1744Cys	N	AD, maternal, het	Inconclusive	Positive	01/2019	02/2020	Follow-up and updated family history	LP (PM1, PM2, PP3, PP4)

**P, epilepsy panel; W, whole-exome sequencing; N, not reported; EE, epileptic encephalopathy; GEFS+, genetic epilepsy with febrile seizures plus; SHE, sleep-related hypermotor epilepsy; RE, Rolandic epilepsy; ID, intellectual disability; EEG, electroencephalograph; MRI, magnetic resonance imaging; GTCS, generalized tonic-clonic seizure; CPSCPS, complex partial seizure; MS, myoclonic seizure; OXC, oxcarbazepine; VPA, valproate; LEV, levetiracetam; TPM, topamax; CBZ, carbamazepine; AD, autosomal dominant; Het, heterozygous. P, Pathogenic; LP, Likely pathogenic; VUS, Variant of unknown significance.**

The results of four patients were downgraded after reinterpretation (P20, P25, P45, and W53). In particular, patients P20 and P25, who presented different epilepsy types, were found to harbor the same inherited missense variant in *RELN* (c.3712A > C, p.Asn1238His), initially rendering the results inconclusive. In the second interpretation, we found that this variant had been submitted to the ClinVar database multiple times and interpreted as Benign as of December 2019. In addition, the MAF of this variant in genomeAD increased from 0.0024 to 0.0075, which was greater than expected for this disorder. The result for case P45 was downgraded also as a result of new information in ClinVar and population databases. For patient W53, an inherited missense variant was detected in *SCN3A* (c.1687C > T, p.Arg563Cys). Through follow-up, this variant was also detected in the patient’s healthy grandmother and younger sister. Moreover, as of January 2020, this variant had been submitted once to the ClinVar database and interpreted as Likely benign (Table 2).

**FIGURE 3:**
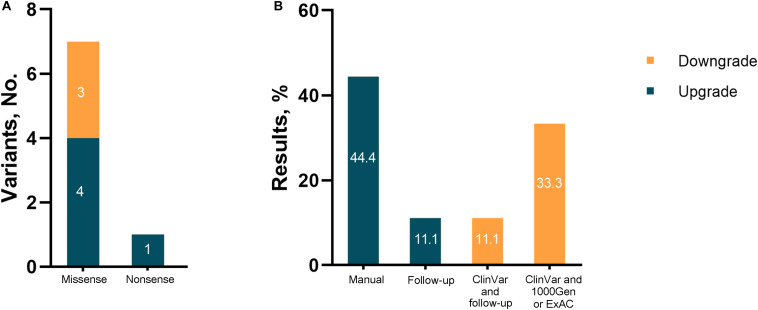
Results reinterpreted in the study. **(A)** Number of reclassified variants; **(B)** evidence for change.

Among the nine revised diagnoses, the most frequently reclassified variants for both upgraded and downgraded yields were missense (7/8, 87.5%), followed by nonsense (1/8, 12.5%). For the upgraded results, diagnostic reclassification was based on a manual analysis involving a literature review or analysis of cases with similar phenotypes (4/5, 80%), and updated information in medical records due to follow-up (1/8, 20%). Inconclusive results were downgraded mainly because of updated information in clinical and population databases (Figure 3).

## Discussion

In the present study, we reported the NGS for 200 consecutive patients with suspected genetic epilepsy included over 3 years. The results highlight the clinical utility of using NGS and the importance of reinterpreting genetic test reports in epilepsy. We identified disease-causing variants in 14.5% of the patients after initial analysis and reinterpretation reports, which is comparable to previous NGS-related reports for epilepsy (Berg et al., 2017; Butler et al., 2017; Perucca et al., 2017). We also showed that NGS can be a valuable tool for detecting the etiology of adult epilepsy. Despite the recent advances in the molecular genetics of this condition, genetic evaluation is often overlooked in adults. In our study, a genetic diagnosis was made in 13 (11%) of 118 adults, which was similar to the diagnostic yield found in pediatric epilepsy (*p* = 0.145). However, our yield was slightly lower than that obtained in previous studies in adults, in which a genetic cause was identified in 22 or 23% of cases, respectively (Borlot et al., 2019; Johannesen et al., 2020). The higher yield in the other studies may have been due to most patients also presenting with ID and/or developmental delay. Although not statistically significant, we noticed that the diagnostic rate is higher in focal than generalized epilepsy (24.5 vs. 7.9%), which is different to what has been described in larger cohort studies (Epi4K consortium and Epilepsy Phenome/Genome Project, 2017; Al-Nabhani et al., 2018). Since our clinic is in a tertiary hospital, which is the largest medical care center in the northwestern China, quite many patients with focal epilepsy whose phenotypes are less severe might just go to local hospitals instead of coming all the way here for further diagnosis and treatment. This may be the reason why patients with focal epilepsy were more likely to accompany with ID than patients with generalized epilepsy in our study (9/49, 18.5% vs. 11/101, 10.9%). Evidence from our study and the studies of some others suggested that epilepsy patients with ID were significantly more likely to have a positive result (Borlot et al., 2019; Johannesen et al., 2020). Thus, at least in part, explain that the diagnostic rate is higher in focal vs. generalized epilepsy in our study.

A key challenge associated with the clinical utility of NGS is data interpretation, especially for variants of uncertain significance (Duzkale et al., 2013; Kassahn et al., 2014; Seaby and Ennis, 2020). Besides, with the rapid advances publicly available databases and the ongoing updating of clinical information of the patients over time, it appears manageable and worthwhile to reinterpret NGS reports for patients with inconclusive genetic test results (Nambot et al., 2018; Epi25 Collaborative, 2019; SoRelle et al., 2019). This strategy allowed us to obtain 9 (10.5%) clinically significant changes in diagnosis among the 86 initially inconclusive results, reinforcing the need for reinterpretation. Among the nine patients with revised cases, five obtained positive diagnoses. This represents a diagnosis rate of 5.8% (5/86) and a marked increase in diagnostic yield overall, which is similar to a recent study (Epilepsy Genetics Initiative) made 5.8% (8/139) new diagnoses during a systematic reanalysis of unresolved WES data (Epilepsy Genetics Initiative, 2019).

The major reason that led to our revised results after reinterpretation was the manual search for additional evidence of pathogenicity for candidate variants and the discovery of new gene-disease relationships. SHE is a focal epilepsy characterized by hypermotor seizures occurring predominantly in clusters during non-rapid eye movement sleep, which is a heterogeneous genetic syndrome, and the transmission pattern of the disorder is compatible with an autosomal dominant inheritance with reduced penetrance (Tinuper et al., 2016). Currently, variants in genes coding for subunits of the heteromeric neuronal nicotinic receptors (nAchR) are the most commonly identified pathogenic variants in SHE (Becchetti et al., 2015). In our SHE patients (P7, W48, and W58), we detected three variants (NM_198903:c.1070C > A, p.Thr357Asn, *de novo*; c.649C > T, p.Gln217X, inherited from unaffected father; c.269C > T, p.Thr90Met, inherited from unaffected mother) in *GABRG2* at different time points (Table 2). Mutations in *GABRG2* are associated with febrile seizures (FS) and various genetic epilepsy syndromes, which suggests a broad range of phenotypical spectrum associated with *GABRG2* variants (Kang and Macdonald, 2016). And an incomplete penetrance has previously been noted for some *GABRG2* mutation-positive families (Johnston et al., 2014). Additionally, by searching and reviewing the corresponding literature, we found that previously reported SHE variants are associated with large selective increases in nicotine-evoked GABAergic inhibition, a likely key factor in epileptogenesis, as the seizures *in vivo* can be blocked by subconvulsive doses of GABA_A_ receptor antagonists (Klaassen et al., 2006). Besides, our experiments on HEK293T cells (unpublished data) have shown that these *GABRG2* variants decrease GABA-evoked currents, and provided further evidence for the pathogenicity of the three *GABRG2* variants in relation to SHE. Combined, these observations suggest that the variants in *GABRG2* are associated with GABAergic disinhibition, thereby promoting SHE epileptogenesis. These variants were not initially reported as pathogenic because of the time lag between these genetic test reports. However, reinterpretation allowed us to re-evaluate these candidate variants over time, which not only benefited the patients, but also expanded the genetic spectrum for epilepsy.

Accurate and comprehensive clinical information was essential for correct and timely diagnosis. Follow-up and updated family segregation data, as in the case of patient W85, allowed us to assign a positive result after reinterpretation. Timely updated information in clinical and population databases also helped uncover new evidence that weakened the support for a particular diagnosis (Table 2: patients P20, P25, P45, and W53). This suggests that a clinician with experience in interpreting genetic diagnoses should take advantage of these resources and periodically perform a systematic review.

This study had several limitations. The patient sample size was relatively small, and there was a possible selection bias as we only enrolled patients who were referred to our Comprehensive Epilepsy Center. Additionally, patients with initially negative results were arbitrarily excluded at reinterpretation owing to unreported variants. This may have led to the absence of an analysis of benign variants that could have been upgraded to pathogenic or uncertain variants.

## Conclusion

In conclusion, we have highlighted the clinical utility of NGS and the importance of reinterpreting inconclusive genetic test reports in epileptic patients. Our results demonstrated the importance of keeping up with expanding knowledge, and stress that patients should be informed that medicine is constantly evolving and genetic test results may change over time. Additionally, our results indicated that routine diagnostic genetic testing should also be considered for adults with epilepsy.

## Data Availability Statement

The datasets for this article are not publicly available due to concerns regarding participant/patient anonymity. Requests to access the datasets should be directed to the WJ, drjiangwen@hotmail.com.

## Ethics Statement

The studies involving human participants were reviewed and approved by the ethics committee of the Xijing Hospital. Written informed consent to participate in this study was provided by the participants’ legal guardian/next of kin. Written informed consent was obtained from the individual(s), and minor(s)’ legal guardian/next of kin, for the publication of any potentially identifiable images or data included in this article.

## Author Contributions

YJ: study concept and design, drafting of the manuscript, critical revision, statistical analysis, and study supervision. CS and YW: genetic analysis and study supervision. JZ, FY, QG, XL, and YM: data acquisition, analysis, and interpretation. WJ: study concept and design, critical revision, and obtained funding. All authors contributed to the article and approved the submitted version.

## Conflict of Interest

The authors declare that the research was conducted in the absence of any commercial or financial relationships that could be construed as a potential conflict of interest.

## Footnotes

1 http://annovar.openbioinformatics.org/

2 http://people.virginia.edu/~wc9c/KING

3 https://search.clinicalgenome.org/kb/gene-validity/72a91ef6-e052-44a4-b14e-6a5ba93393ff–2018-06-15T18:23:54
