# Supplementary material for: Clinical Utility of Exome Sequencing and Reinterpreting Genetic Test Results in Children and Adults With Epilepsy
Source: Front Genet. 2020 Dec 18;11:591434. doi: 10.3389/fgene.2020.591434 (PMC7775549; doi:10.3389/fgene.2020.591434)
Supplement: Supplementary file 1 [file Table_1.DOC]

Supplementary Table 1. The epilepsy gene panel

| ADSL | CHD2 | DHFR | GLB1 | MAGI2 | PNPO | SLC9A6 |
| --- | --- | --- | --- | --- | --- | --- |
| ALDH7A1 | CHRNA2 | DIAPH3 | GLRA1 | MAPK10 | POLG | SPTAN1 |
| ALG13 | CHRNA4 | DNAJC6 | GPR56 | MBD5 | PPT1 | SRPX2 |
| ARG1 | CHRNA7 | DNM1 | GPR98 | MDGA2 | PROC | ST3GAL2 |
| ARHGEF15 | CHRNB2 | DOCK7 | GRIN1 | ME2 | PRRT2 | ST3GAL5 |
| ARHGEF9 | CLCN2 | EEF1A2 | GRIN2A | MECP2 | RBFOX1 | STRADA |
| ARX | CLCN4 | EFHC1 | GRIN2B | MEF2C | RBFOX2 | STXBP1 |
| ASAH1 | CLN3 | ELP4 | HAX1 | MFSD8 | RBFOX3 | SYNGAP1 |
| ATP13A4 | CLN5 | EPHB2 | HDAC4 | MTHFR | RELN | SYNJ1 |
| ATP1A2 | CLN6 | ERBB4 | HEXA | MTOR | RYR3 | SZT2 |
| ATP1A3 | CLN8 | FASN | HEXB | NDE1 | SCN1A | TBC1D24 |
| ATP6AP2 | CNTN5 | FLNA | HNRNPH1 | NEDD4L | SCN1B | TCF4 |
| ATP7A | CNTNAP2 | FOLR1 | HNRNPU | NID2 | SCN2A | TNK2 |
| BRAF | COX6B1 | FOXG1 | IQSEC2 | NRXN1 | SCN8A | TPP1 |
| BSN | CSTB | FOXP2 | KCNB1 | PAFAH1B1 | SHANK3 | TSC1 |
| CACNA1A | CTNNA3 | GABBR2 | KCNH5 | PCDH19 | SLC13A5 | TSC2 |
| CACNA1H | CTSD | GABRA1 | KCNMA1 | PDHA1 | SLC19A3 | TUBA1A |
| CACNB4 | CYB5R3 | GABRA6 | KCNQ2 | PIGA | SLC1A3 | UBE3A |
| CASK | DBH | GABRB2 | KCNQ3 | PIGV | SLC25A22 | VRK2 |
| CDH13 | DCX | GABRB3 | KCNT1 | PLCB1 | SLC2A1 | WDR45 |
| CDH9 | DEPDC5 | GABRD | LGI1 | PNKD | SLC35A2 | ZEB2 |
| CDKL5 | DGKD | GABRG2 | LIAS | PNKP | SLC46A1 |  |
