# Supplementary material for: Clinical Utility of Exome Sequencing and Reinterpreting Genetic Test Results in Children and Adults With Epilepsy
Source: Front Genet. 2020 Dec 18;11:591434. doi: 10.3389/fgene.2020.591434 (PMC7775549; doi:10.3389/fgene.2020.591434)
Supplement: Supplementary file 2 [file Table_2.DOC]

Supplementary table 2.

|  | Gene-panel | WES |
| --- | --- | --- |
| Paired-end read length | 150bp | 150bp |
| Raw_data_bases(Mb) | 3977.99 | 16151.84 |
| Clean_data_bases(Mb) | 3739.01 | 15767.11 |
| Aligned_bases(Mb) | 3720.40 | 15761.96 |
| Aligned | 99.27% | 97.57% |
| Initial bases on target | 1243686.06 | 49109808 |
| Base covered on target | 1238323.54 | 48839285.04 |
| Coverage of target region | 99.20% | 99.45% |
| Effective bases on target | 433146142.01 | 7863204293.22 |
| Fraction of effective bases on target | 14% | 0.56 |
| Average sequencing depth on target | 334.50 | 160.11 |
| Fraction of target covered with at least 4X | 98.44% | 99.02% |
| Fraction of target covered with at least 10X | 97.50% | 98.53% |
| Fraction of target covered with at least 20X | 95.71% | 97.41% |
| duplication rate | 23.4% | 10.51% |
