# Supplementary material for: Clinical Utility of Exome Sequencing and Reinterpreting Genetic Test Results in Children and Adults With Epilepsy
Source: Front Genet. 2020 Dec 18;11:591434. doi: 10.3389/fgene.2020.591434 (PMC7775549; doi:10.3389/fgene.2020.591434)
Supplement: Supplementary file 3 [file Table_3.DOC]

Supplementary Table 3. Clinical and genetic characteristics of patients with positive results following the initial analysis and reinterpretation

| Case | Gender/age studied, y | Gene | Variant details | Variant type | Inheritance | Zygosity | References | Patient Phenotype | Family history* | ACMG | Published Phenotype (OMIM or HGMD #) |
| --- | --- | --- | --- | --- | --- | --- | --- | --- | --- | --- | --- |
| Initial analysis | | | | | | | | | | | |
| P1 | M/5 | PRRT2 | NM_145239  c.641dupC  p.Ala214fs | Frameshift | AD, Paternal | Het | Chen,et al. 2011 | ICCA (GTCS; seizure onset 1 year; seizure-free for 3 years on VPA) | FS in father | P (PVS1, PM2) | Episodic kinesigenic dyskinesia 1  (#128200) |
| P8 | F/15 | CHRNA4 | NM_000744 c.979G>A p.Val327Met | Missense | AD, Paternal | Het | Jiang, et al. 2018 | GEFS+ (GTCS+MS; onset 3 years; seizure-free for 4 years on LEV) | PKD in father and young brother | LP (PS4, PM1, PM2, PP3) | Epilepsy, nocturnal frontal lobe, 1; ENFL1 (#600513) |
|  |  | CHRNB2 | NM_000748 c.1381C>T p.Leu461Phe | Missense |  | Het |  |  |  | VUS (PM1, PP3) | Epilepsy, nocturnal frontal lobe, 3  (#605375) |
| P13 | F/25 | ADGRV1 | NM_032119  c.7945+1G>T Splicing | Splicing | AD, Paternal | Het |  | GEFS+ (GTCS+CPS; onset 2 years; intractable on LTG+VPA) |  | LP (PVS1, PM2) | Febrile seizures, familial, 4  (#604352) |
| P22 | M/16 | CHRNA4 | NM_000744  c.1007G >A p.Arg336His | Missense | AD, Paternal | Het | Chen,et al. 2009; Weltzin MM, et al. 2015 | SHE (onset 16 years; seizure-free for 4 years on OXC; sharp and slow complex wave at the left frontal on EEG), previous history of FS starting at 4 year | SHE in young sister | P (PS3, PS4, PM1, PP4) | Epilepsy, nocturnal frontal lobe, 1; ENFL1 (#600513) |
| P36 | M/19 | SCN1A | NM_006920  c.2207T>C  p.Ile736Thr | Missense | AD,  *De novo* | Het |  | MS (onset 13 years; seizure-free for 5 years on VPA and LEV), spike and slow wave complex at the temporal, parietal and occipital lobes on EEG, ID |  | LP (PS2, PM2, PP3, PP4) | Epilepsy, generalized, with febrile seizures plus, type 2  (#604403) |
| P41 | F/28 | FLNA | NM_001110556  c.3859_3860del  p.Glu1287fs | Frameshift | XD, Unclear | Het |  | GTCS+CPS (onset 15 years; intractable on LTG+VPA+CBZ+TPM), spike-wave on EEG, gray matter heterotopia on MRI, ID |  | P (PVS1, PM2, PP4) | Heterotopia, periventricular, 1  (#300049) |
| P46 | F/9 | WDR45 | NM_007075  c.19C>T  p.Arg7X | Nonsense | XD,  *De novo* | Het | Haack,et al. 2012; Hamdan FF,et al. 2014; Chen H, et al. 2014 | EE (GTCS; onset 2 years; seizure-free for 5 years on VPA), sharp-wave and spike-slow waves at the frontal frontal lobe, ID, language developmental delay |  | P (PVS1, PS2, PS4, PM2) | Neurodegeneration with brain iron accumulation 5  (#300894) |
| P50 | F/8 | TSC2 | NM_000548  c.2220+1G>A  Splicing | Splicing | AD,  *De novo* | Het |  | TSC (GTCS+CPS; onset 1 years; intractable on LTG+VPA+TPM; spike and slow wave complex at the frontal, temporal and occipital lobes on EEG; bilateral periventricular multiple nodules on MRI), ID |  | P (PVS1, PS2, PM2) | Tuberous sclerosis-2  (#613254) |
| P71 | M/2 | SCN1A | NM_006920  c.4522C>A  p.Pro1508Thr | Missense | AD,  *De novo* | Het | Moehring J, et al. 2013 | Dravet syndrome (CPS+GTSC+MS; onset 1 year; intractable on VPA+LEV; spike and slow wave complex on EEG), ID |  | P (PS2, PS4, PM1, PM2, PM5, PP3, PP4) | Epileptic encephalopathy, early infantile, 6 (Dravet syndrome)  (#607208) |
| P74 | F/15 | GABRA1 | NM_000806  c.640C>T  p.Arg214Cys | Missense | AD,  *De novo* | Het | Demos M, et al. 2019 | GTCS+CPS (onset 11 years; intractable on TPM+LTG; sharp and slow wave complex on EEG), autistic features, and intermittent aggressive behavior, ID |  | P (PS2, PS4, PM1, PM2, PM5, PP3) |  |
| P76 | F/15 | SLC2A1 | NM_006516  c.635G>A  p.Arg212His | Missense | AD, Maternal | Het | Klepper,et al., 2005 | EE (GTCS+CPS+atonic seizure; onset 2 years; intractable on LEV+VPA+LTG), spike-slow waves at the forehead on EEG, ID |  | LP (PM1, PM2, PP3, PP4, PP5) | GLUT1 deficiency syndrome 1, infantile onset, severe  (#606777) |
| W11 | M/26 | LGI1 | NM_005097 c.1238G>A p.Trp413X | Nonsense | AD, Paternal | Het |  | sGTCS+auditory symptoms (onset 24 years; seizure-free for 2 years on OXC) | TLE in father | P (PVS1, PM2, PP3, PP4) | Epilepsy, familial temporal lobe (#600512) |
| W12 | F/28 | LGI1 | NM_005097 c.535T>C p.Cys179Arg | Missense | AD, Maternal | Het | Di Bonaventura,et al., 2011 | CPS+auditory symptoms (onset 7 years; seizure- free for 5 years on LTG) | FS in mother | P (PS4, PM1, PM2, PP3, PP4) | Epilepsy, familial temporal lobe (#600512) |
| W19 | M/15 | CHRNB2 | NM_000748  c.842T>G  p.Leu281Arg | Missense | AD,  *De novo* | Het |  | SHE (onset 2 years; intractable on LEV+OXC; spike-wave at frontal lobe on EEG), ID |  | LP (PS2, PM2, PP3, PP4) | Epilepsy, nocturnal frontal lobe, 3  (#605375) |
| W20 | M/25 | SCN1A | NM_001165963.1  c.5293T>C  p.Phe1765Leu | Missense | AD, Paternal | Het |  | GEFS+ (sGTCS, onset 1 year; intractable on LEV+OXC) | FS/FS+ in father, grandfather, male/female cousin | LP (PM1, PM2, PM5, PP1, PP3, PP4) | Epilepsy, generalized, with febrile seizures plus, type 2  (#604403) |
| W50 | M/24 | ACADS | NM_000017  c.578C>T  p.Ser193Leu | Missense | AR, Paternal | Compound  het |  | MS (seizure onset 17 years; seizure-free for 2 years on LEV), spike-slow waves on EEG, ethylmalonic acid concentrations elevated in urinary urinary biochemical examinations |  | LP (PM1, PM2, PM3, PP3) | Acyl-CoA dehydrogenase, short-chain, deficiency of  (#201470) |
|  |  |  | NM_000017  c.1156C>T  p.Arg386Cys | Missense | AR, Maternal |  | Merinero,et al., 2006 |  |  | LP (PM1, PM2, PM3, PP3) |  |
| W92 | M/20 | CHRNA2 | NM_000742.3  c.1250T>A p.Val417Glu | Missense | AD, Maternal | Het | Chen,et al. 2011 | SHE (onset 17 years; intractable on LTG; epileptiform wave at the prefrontal lobe on EEG) | Epilepsy in mother, maternal's brother and father | LP (PM1, PM2, PP1, PP4, BP4) | Epilepsy, nocturnal frontal lobe, 4，ENFL4 (#610353) |
| W95 | F/20 | LGI1 | NM_005097  c.1619_1624delGTTTTA  p.S540_F541del | Frameshift | AD,  *De novo* | Het |  | TLE (onset 15 years; seizure-free for 4 years) |  | LP (PS2, PM2, PM4, PP4) | Episodic kinesigenic dyskinesia 1  (#128200) |
| W118 | M/22 | DEPDC5 | NM_001136029  c.3967C>T  p.Arg1323Ter | Missense | AD,  *De novo* | Het | Ricos,et al., 2016 | GTCS+CPS (onset 17 years; intractable on LEV), ID |  | P (PVS1, PS2, PM2, PP3) | Epilepsy, familial focal, with variable foci 1 (#604364) |
| W119 | M/15 | GRIN2B | NM_000834.3  c.1559C>T  p.Ser520Leu | Missense | AD, Maternal (FS) | Het |  | MS+CTCS (seizure onset 3 days after birth; intractable on LEV+VPA), spike-slow burst waves on EEG, ID, his Maternal and Maternal’s Paternal have epilepsy | FS in mother; Epilepsy in maternal’s father | LP (PM1, PM2, PP1, PP4) | Epileptic encephalopathy, early infantile, 27  (#616139) |
| W127 | M/8 | GABRD | NM_000815  c.1108G>A  p.Val370Ile | Missense | AD, Maternal | Het |  | GEFS+ (GTCS; onset 2 years; seizure-free for 2 years on LEV; sharp and slow wave complex on EEG) | FS/FS+ in mother and his brother | LP (PM1, PM2, PP1,PP4, BP4) | Epilepsy, generalized, with febrile seizures plus, type 5, susceptibility to  (#613060) |
| W128 | M/7 | TSC2 | NM_000548  c.3397+5G>A  Splicing | Splicing | AD,  *De novo* | Het | Choy,et al., 2009 | TSC (CPS; onset 3 years; intractable on LEV+OXC), ID |  | P (PS2, PS4, PM2, PP4) | Tuberous sclerosis-2  (#613254) |
| W132 | M/40 | TSC2 | NM_000548.4  c.1443+5G>C  Splicing | Splicing | AD,  *De novo* | Het | Suspitsin,et al., 2018 | TSC (GTCS+ gelastic seizure; onset 2 years; intractable on LEV+OXC; multiple abnormal signals at the bilateral hemisphere on MRI) |  | P (PS2, PS4, PM2, PP4) | Tuberous sclerosis-2  (#613254) |
| W133 | M/14 | SCN1A | NM_001165963  c.5020G>A  p.Gly1674Ser | Missense | AD, Paternal | Het | Saitoh,et al., 2015 | GEFS+ (GTCS+CPS; onset 1 year; intractable on OXC+VPA) | FS in father | P (PS4, PM1, PM2, PM5, PP3, PP4) | Epilepsy, generalized, with febrile seizures plus, type 2  (#604403) |
| Reinterpretation | | | | | | | | | | | |
| P5 | M/10 | GRIN2A | NM_000833 c.1341T >A p.Asn447Lys | Missense | AD, Maternal | Het | Xu,et al., 2017 | RE (CPS+sGTCS; onset 8 years; intractable on LEV +VPA), centro-temporal spikes on EEG |  | P (PS3, PS4, PM1, PM2, PP4, BP4) | Epilepsy, focal, with speech disorder and with or without mental retardation  (#245570) |
| P72 | M/12 | GABRG2 | NM_198903  c.1070C>A  p.Thr357Asn | Missense | AD,  *De novo* | Het |  | SHE (onset 7 years; intractable on OXC), ID |  | LP (PS2, PM1, PM2, PP3) | Epilepsy, generalized, with febrile seizures plus, type 3  (#607681) |
| W48 | M/36 | GABRG2 | NM_198903  c.649C>T  p.Gln217X | Nonsense | AD, Paternal | Het |  | SHE (onset 12 years; seizure-free for 2 years on CBZ; rare sharp-slow wave at the forehead during sleep) |  | LP (PVS1, PM2) |  |
| W51 | F/9 | GABRG2 | NM_198903  c.269C>T  p.Thr90Met | Missense | AD, Maternal | Het |  | SHE (onset 7 years; Some improvement on CBZ; 3.5-4Hz spike-slow wave at the forehead and anterior temporal) |  | LP (PM1, PM2, PP3, PP5) |  |
| W85 | M/33 | SCN9A | NM_002977.3  c.5231A>G p.Tyr1744Cys | Missense | AD, Maternal | Het |  | GTCS, onset 8 years, intractable on LEV | FS in mother; GEFS+ in his younger sister | LP (PM1, PM2, PP3, PP4) | Epilepsy, generalized, with febrile seizures plus, type 7  (#613863) |

*: The following family members also carrying variant which identified in the proband detected by Sanger sequencing.

Abbreviation: P, epilepsy panel; W: whole exome sequencing; M, male; F, female; AD, autosomal dominant; AR, autosomal recessive; XD, X-linked dominant; Het, heterozygous; ICCA, Infantile convulsions and paroxysmal choreoathetosis; PKD, Paroxysmal kinesigenic dyskinesia; EE, Epileptic encephalopathy; GEFS+, Genetic epilepsy with febrile seizures plus; FS, Febrile seizure; FS+, Febrile seizure plus; TLE, Temporal lobe epilepsy; SHE, Sleep-related hypermotor epilepsy; RE, Rolandic epilepsy; TSC, Tuberous sclerosis; ID, Intelligence disability; EEG, Electroencephalograph; MRI, Magnetic Resonance Imaging; GTCS, Generalized tonic-clonic seizure; sGTCS, secondary GTCS; CPS, Complex partial seizure; MS, Myoclonic seizure; OXC, Oxcarbazepine; VPA, Valproate; LEV, Levetiracetam; TPM, Topamax; LTG, lamotrigine; CBZ, carbamazepine; P, Pathogenic; LP, Likely pathogenic; VUS, Variant of unknown significance.
