# Supplementary material for: Clinical Utility of Exome Sequencing and Reinterpreting Genetic Test Results in Children and Adults With Epilepsy
Source: Front Genet. 2020 Dec 18;11:591434. doi: 10.3389/fgene.2020.591434 (PMC7775549; doi:10.3389/fgene.2020.591434)
Supplement: Supplementary file 4 [file Table_4.DOC]

Supplementary Table 4 Secondary findings

| Case | Gene | Variant details | Variant type | Inheritance | Zygosity | Patient Phenotype | ACMG | Published Phenotype (OMIM or HGMD #) |
| --- | --- | --- | --- | --- | --- | --- | --- | --- |
| W72 | TGFBR2 | NM_003242.5  c.95-2A>G  splicing | splicing | AD | Het | GTCS (onset 2 years; intractable on LEV), spike-slow waves on EEG, ID | LP | Loeys-Dietz syndrome 2  (#610168) |
| W110 |  |  |  |  |  | GTCS+MS (onset 13 years; seizure free for 2 years on LTG), spike waves on EEG |  |  |
| W98 | LDLR | NM_000527.4  c.268G>A p.Asp90Asn | Missense | SD | Het | CPS (onset 1 year; intractable on VPA+TPM+CBZ) | LP | Hypercholesterolemia, familial, 1 (#143890) |

Abbreviation: AD, autosomal dominant; SD, Semidominant; Het, heterozygous. GTCS, Generalized tonic-clonic seizure; CPS, Complex partial seizure; MS, Myoclonic seizure; VPA, Valproate; LEV, Levetiracetam; TPM, Topamax; LTG, lamotrigine; CBZ, carbamazepine; LP, Likely pathogenic.
