# Supplementary material for: Clinical Utility of Exome Sequencing and Reinterpreting Genetic Test Results in Children and Adults With Epilepsy
Source: Front Genet. 2020 Dec 18;11:591434. doi: 10.3389/fgene.2020.591434 (PMC7775549; doi:10.3389/fgene.2020.591434)
Supplement: Supplementary file 5 [file Table_5.DOC]

Supplementary Table 5. The frequency of variants in gnomAD and local datasets

| Gene | Variant details | Frequency | | Gene | Variant details | Frequency | | |
| --- | --- | --- | --- | --- | --- | --- | --- | --- |
|  |  | gnomAD | Local datasets |  |  | gnomAD | | Local datasets |
| PRRT2 | NM_145239  c.641dupC  p.Ala214fs | 0.0002 | 0.00132 | CHRNB2 | NM_000748  c.842T>G  p.Leu281Arg | Absent | Absent | |
| CHRNA4 | NM_000744 c.979G>A p.Val327Met | Absent | Absent | SCN1A | NM_001165963.1  c.5293T>C  p.Phe1765Leu | Absent | Absent | |
| CHRNB2 | NM_000748 c.1381C>T p.Leu461Phe | 0.00003233 | Absent | ACADS | NM_000017  c.578C>T  p.Ser193Leu | Absent | Absent | |
| ADGRV1 | NM_032119  c.7945+1G>T Splicing | Absent | Absent |  | NM_000017  c.1156C>T  p.Arg386Cys | 0.00003189 | Absent | |
| FLNA | NM_001110556  c.3859_3860del  p.Glu1287fs | Absent | Absent | CHRNA2 | NM_000742.3  c.1250T>A p.Val417Glu | Absent | Absent | |
| WDR45 | NM_007075  c.19C>T  p.Arg7X | Absent | Absent | LGI1 | NM_005097  c.1619_1624delGTTTTA  p.S540_F541del | Absent | Absent | |
| TSC2 | NM_000548  c.2220+1G>A  Splicing | Absent | Absent | DEPDC5 | NM_001136029  c.3967C>T  p.Arg1323Ter | Absent | Absent | |
| SCN1A | NM_006920  c.4522C>A  p.Pro1508Thr | Absent | Absent | GRIN2B | NM_000834.3  c.1559C>T  p.Ser520Leu | Absent | Absent | |
| GABRA1 | NM_000806  c.640C>T  p.Arg214Cys | Absent | Absent | GRIN2A | NM_000833.3  c.3619T>C p.Tyr1207His | Absent | 0.00022 | |
| SLC2A1 | NM_006516  c.635G>A  p.Arg212His | Absent | Absent | GABRD | NM_000815  c.1108G>A  p.Val370Ile | Absent | Absent | |
| LGI1 | NM_005097 c.1238G>A p.Trp413X | Absent | Absent | SCN3A | NM_006922  c.1687C>T  p.Arg563Cys | 0.0002 | 0.00022 | |
| LGI1 | NM_005097 c.535T>C p.Cys179Arg | Absent | Absent | DEPDC5 | NM_001242897  c.2858C>A p.Pro953His | 0.0004 | 0.00594 | |
| TSC2 | NM_000548  c.3397+5G>A  Splicing | Absent | Absent | SCN1A | NM_001165963  c.5020G>A  p.Gly1674Ser | Absent | Absent | |
| TSC2 | NM_000548.4  c.1443+5G>C  Splicing | Absent | Absent | SCN9A | NM_002977.3  c.5231A>G p.Tyr1744Cys | Absent | Absent | |
| GABRG2 | NM_198903  c.1070C>A  p.Thr357Asn | Absent | Absent | RELN | NM_005045.3  c.3712A>C  p.Asn1238His | 0.0074 | 0.00594 | |
| GABRG2 | NM_198903  c.649C>T  p.Gln217X | Absent | Absent | CHRNA4 | NM_000744  c.1007G >A p.Arg336His | 0.00003187 | 0.00022 | |
| GABRG2 | NM_198903  c.269C>T  p.Thr90Met | Absent | Absent | SCN1A | NM_006920  c.2207T>C  p.Ile736Thr | Absent | 0.00022 | |
